# Case Report: Right−sided vagus nerve stimulation for treatment−resistant affective disorders with complex cardiac comorbidity

**DOI:** 10.3389/fpsyt.2026.1754397

**Published:** 2026-03-19

**Authors:** Christiane Licht, Michael Landgrebe, Tom Pieper

**Affiliations:** 1 Department of Psychiatry, Psychotherapy and Psychosomatics, Rheinisch-Westfälische Technische Hochschule (RWTH) University Clinic, Aachen, Germany; 2 Department of Psychiatry and Psychotherapy, University of Regensburg, Regensburg, Germany; 3 Department of Psychiatry, Psychosomatics and Psychotherapy, kbo-Lech-Mangfall-Klinik Agatharied, Hausham, Germany; 4 Department of Psychiatry, Psychotherapy and Psychosomatics, Clinic Wilhelmshaven, Wilhelmshaven, Germany

**Keywords:** bipolar disorder, case report, case series, pacemaker VNS, right-sided VNS, treatment-resistant depression, vagus nerve stimulation, VNS

## Abstract

**Background:**

Vagus nerve stimulation (VNS) is a well-established treatment option for treatment-resistant affective disorders. Due to potential concerns about increased cardiac innervation on the right side, devices are almost exclusively implanted on the left cervical vagus nerve. Clinical data on right-sided VNS for psychiatric indications are often very scarce.

**Methods:**

We present a case series involving three patients—two with MDD and one with bipolar depression—each with extensive prior unsuccessful treatments, frequent hospitalizations, and severe psychiatric and medical comorbidities. In each case, the right cervical side was chosen for primary implantation or re-implantation due to cardiac disease, pre-existing devices, or anatomical constraints (including high-grade atrioventricular block with pacemaker and recurrent laryngeal nerve palsy). All patients underwent standard titration of stimulation parameters and long-term clinical follow-up.

**Results:**

All three patients showed significant and lasting mood stabilization within approximately 12 months after right-sided VNS implantation. Hospital admission rates decreased markedly, and in two cases, maintenance electroconvulsive therapy (ECT) was discontinued or notably reduced. In all cases, VNS was generally well tolerated. During right-sided stimulation, no clinically significant bradyarrhythmia, device interactions, or other serious cardiac adverse events were observed.

**Conclusions:**

This series highlights the usefulness of right-sided VNS in sustaining long-term mood stability and reducing hospitalizations for patients with highly treatment-resistant, comorbid mood disorders. The findings expand existing epilepsy-focused data on right-sided VNS to the psychiatric field and support systematic evaluation of this approach in future prospective studies. Multidisciplinary coordination is essential to minimize risks and improve outcomes. Additional long-term research is needed to assess its lasting effectiveness, optimal candidate selection, and cost-efficiency in routine clinical practice.

## Introduction

Depressive disorders are among the most common and severe mental illnesses worldwide. They contribute significantly to the global burden of disease and mortality. Currently, approximately 280 million people are affected by Major Depressive Disorder (MDD), and 50 million by bipolar disorder (1). Despite advances in pharmacological and non-pharmacological treatments, including psychotherapy, neurostimulation like (ECT) or repetitive transcranial magnetic stimulation (rTMS), many patients are considered treatment-resistant, meaning they do not achieve sufficient improvement or remission even after several adequate trials with antidepressants or psychotherapy. For these individuals, vagus nerve stimulation (VNS) has emerged as an effective treatment option with promising results for patients refractory to drugs and psychotherapies (2).

VNS generally involves intermittent electrical stimulation of the left vagus nerve. Several clinical trials and naturalistic case series indicate that this approach may support long-term mood stability, higher response and remission rates, and a reduction or discontinuation of maintenance ECT (3) (4). Regulatory approvals and surgical practices have been primarily influenced by the belief that the right vagus nerve has a greater impact on cardiac function, especially on the sinoatrial node, raising concerns about bradyarrhythmia and asystole during right-sided stimulation. Consequently, left-sided implantation has become the standard in both epilepsy and psychiatry. However, much of the evidence regarding different cardiac risks is based on animal studies rather than extensive human data (5).

In contrast, clinical experience with right-sided VNS remains limited. A recent multicenter survey collected 38 cases of right-sided VNS and found seizure reductions comparable to left-sided stimulation, with no life-threatening cardiac complications and a safety profile mainly characterized by transient stimulation-related side effects such as hoarseness or dyspnea (5).

Although these data come from epilepsy populations and primarily focus on seizure control and device safety, they provide important preliminary information on the cardiac risk profile of right-sided stimulation and its technical feasibility in humans.

These findings challenge the traditional view that right-sided VNS is inherently more dangerous and suggest that, in selected situations, implantation on the right cervical vagus nerve can be a clinically reasonable alternative.

Regarding psychiatric indications, however, there is little systematic data on right-sided VNS, even though patients with treatment-resistant affective disorders often present with complex somatic comorbidities, cardiac devices, or anatomical constraints that may restrict left-sided surgery. In such cases, right-sided VNS may serve as an essential option to provide neuromodulatory treatment while reducing surgical and cardiovascular risks.

Below, we present a case series of three patients with treatment-resistant affective disorders and significant psychiatric and somatic comorbidities who all underwent right-sided VNS implantation after extensive prior treatment.

In addition to the individual case descriptions, we systematically outline the clinical selection criteria that led to right-sided implantation rather than the conventional left-sided approach, describe cardiological and anatomical risk considerations, and discuss how these experiences may inform candidate selection and multidisciplinary decision-making for right-sided VNS in routine psychiatric practice.

The aim is to illustrate the illness progression, clinical benefits, potential complications, challenges, and discuss the implications for right-sided VNS in everyday psychiatric practice amid emerging evidence in neuromodulation research.

## Case description

Here, we present three patients diagnosed with MDD (N = 2) and bipolar depression (N = 1) who were treated with right-sided VNS. For all three patients, the decision to proceed with implantation was discussed at a multidisciplinary team meeting involving neurosurgeons, neurologists, and psychiatrists.

In our clinical setting, right-sided VNS was considered only when standard left-sided implantation was contraindicated or carried an unacceptable risk profile, as determined by cardiological or anatomical assessment. Relevant factors included pre-existing cardiac conduction abnormalities, prior left-sided cardiac surgery or device implantation, concerns about potential device–device interactions, and a history of recurrent laryngeal nerve palsy with an increased risk of bilateral vocal cord dysfunction. During the observation period, no patients with similar profiles underwent left-sided VNS rather than right-sided implantation; patients who declined VNS altogether were excluded from this series.

All patients underwent preoperative cardiological evaluation, including ECG and, when indicated, echocardiography to assess conduction abnormalities and structural heart disease. Perioperatively and during initial titration, continuous ECG or telemetry monitoring was used to detect potential bradyarrhythmias or other stimulation-related cardiac events. During follow-up, patients received regular cardiology checkups alongside psychiatric assessments, with device interrogation as needed.

Here, the term “remission” refers to a sustained absence of full syndromal criteria for a major depressive or bipolar depressive episode, typically corresponding to minimal or mild symptom levels on routine clinical assessment and available rating scales. The term “partial remission” denotes a marked reduction in symptom severity and functional impairment without complete resolution of affective symptoms.

### Case 1: remission 1 year after right-sided implantation

The first patient is an 85-year-old woman with a long history of recurring depressive episodes, first diagnosed in 1998. Over the years, she has undergone multiple extended inpatient psychiatric treatments, during which several suicide attempts occurred, including one while she was hospitalized. Notably, her mother died by suicide after experiencing bereavement, indicating a family history of mood disorders.

After receiving a wide range of pharmacotherapies, including selective serotonin reuptake inhibitors (Citalopram, Mirtazapine), monoamine oxidase inhibitors (Moclobemide), tricyclic antidepressants (Amitriptyline, Tetracyclics intravenously, Venlafaxine), antipsychotics (Haloperidol, Olanzapine, Risperidone, Quetiapine), benzodiazepines (Lorazepam, Diazepam, Clonazepam), anticonvulsants (Carbamazepine, Lamotrigine), lithium, and memantine, no remission was achieved. Adjunctive treatments such as thyroid hormone augmentation, “drug holidays,” light therapy, and rTMS (two courses), along with nutritional support, did not improve the depressive symptoms.

Two series of ECT were given but had to be stopped because the patient developed a delirious state.

In 2003, a left-sided VNS (Pulse Model 102) was implanted, with remission occurring in 2004. After a routine generator replacement in 2012, which resulted in a severe wound healing disorder and subsequent right-sided recurrent nerve paralysis, a right-sided VNS (Pulse Model 102) was implanted in 2023 (see **Table 1** for stimulation parameters).

**Table 1:** VNS parameters for each patient, patient 1: initial left−sided implantation in 2003, right−sided re−implantation in 2023; Patients 2 and 3: primary right−sided implantation.

Patient	Intensity (mA)	Frequency(Hz)	Pulse width(ms)	On time (sec)	Off time (min	SideofVNS	Diagnosis
1	"2.25	20	250	30	8	2003: left	MDD
						2023: right	
2	"'1.75	25	500	21	5	right	MDD
3	"'1.75	20	250	21	8	right, pacemaker left	bipolar depression

She achieved complete remission of her symptoms again in 2024, allowing the gradual withdrawal of all psychiatric medications except lithium. To date, no clinically significant bradyarrhythmia, device interactions, or other serious cardiac adverse events have been observed.

### Case 2: remission after right-sided VNS implantation

The patient is a 45-year-old woman who presented in 2018 with persistent depressive symptoms. Her psychiatric history includes recurrent episodes of depression, posttraumatic stress disorder, and a complex somatoform pain disorder. She also exhibits significant personality pathology and has a background of a highly stressful family environment, including experiences of abuse during childhood and adolescence. There is a notable family psychiatric history, with her father diagnosed with narcissistic personality disorder and a sister likely suffering from schizoaffective disorder.

Her medical history includes the surgical removal of a brain tumor (plexus papilloma) in 1992, followed by intensive treatments and multiple complications. In 1994, she developed right-sided recurrent laryngeal nerve palsy.

Due to her chronic pain (migraines attacks 3–4 times a month) and depressive symptoms, she initially tried intravenous ketamine therapy, which proved ineffective. She then underwent a series of 10 ECT sessions, resulting in a significant remission of depression and cessation of tramadol use for pain. Maintenance ECT was administered, but she developed persistent cognitive deficits that strongly affected her performance as a nurse.

Due to the right-sided recurrent laryngeal nerve palsy that developed in 1994 and the associated risk of bilateral recurrent laryngeal nerve damage, the VNS was implanted on the right side.

In 2018, after VNS implantation (DeimpulseDuo Model 103; see **Table 1** for stimulation parameters), she experienced significant stabilization and no longer required hospitalizations.

Since the implantation, depressive crises have been effectively managed on an outpatient basis, allowing her to resume and maintain her professional activities since 2019. To date, no clinically significant bradyarrhythmia, device interactions, or other serious cardiac adverse events have been observed.

### Case 3: remission after right-sided VNS in bipolar depression

This patient is a 73-year-old woman with a longstanding diagnosis of bipolar affective disorder, primarily with depressive episodes, who has been receiving regular psychiatric care at our outpatient department since August 2019. Her medical history includes several severe somatic comorbidities, such as third-degree AV block with a left-sided cardiac pacemaker implanted in 2016, arterial hypertension, hyperthyroidism, insulin-dependent type II diabetes mellitus, osteoporosis, chronic kidney disease secondary to prolonged lithium therapy from 1993 to 2014, and a history of oophorectomy.

Due to declining kidney function, her long-term lithium treatment was discontinued, resulting in repeated, prolonged hospital stays for severe depressive episodes that kept relapsing. Efforts to restart lithium were unsuccessful due to worsening kidney function and a subsequent lithium overdose. In August 2019, she was admitted for electroconvulsive therapy (ECT) after experiencing a catatonic state with severe psychomotor impairment, delusions of relationship, and incontinence. During this episode, 13 ECT sessions were administered, resulting in significant improvements in her mood and cognitive function, as reflected by her MoCA score increasing from 17 to 28 points. She then continued with maintenance ECT, but logistical challenges related to her geographic location limited its long-term effectiveness.

Given recurrent partial remissions and the impracticality of ongoing ECT, she underwent VNS implantation in 2020. During the first few months after implantation, she received maintenance ECT approximately every four weeks. Later, the patient achieved a stable, partially remitted state, with symptoms well-controlled enough to avoid further inpatient treatments. Her psychiatric medication regimen has included mirtazapine, aripiprazole, milnacipran, and various adjunctive therapies tailored to her medical co-morbidities.

With right-sided VNS (DemipulseDuo Model 103; see **Table 1** for stimulation parameters), the patient remained clinically stable and experienced a significant reduction in hospitalizations. Due to the imminent depletion of the generator’s battery and considering the previous course after stopping lithium and subsequent decompensation, an elective generator replacement was scheduled to prevent clinical deterioration. Given her complex medical and psychiatric history, VNS remains a vital part of her long-term treatment plan.

## Discussion

This case series further illustrates that right-sided vagus nerve stimulation (VNS) can be highly effective for patients with severe, treatment-resistant mood disorders.

Especially, because much of the existing evidence on right−sided VNS safety and cardiac effects has been generated in epilepsy populations, while mood changes are usually secondary or not systematically assessed. By contrast, our report focuses specifically on psychiatric indications, extending the largely epilepsy−based safety data on right−sided VNS to patients with treatment−resistant affective disorders.”

It also highlights the complexity of selecting patients for VNS in real-world clinical practice. All three patients experienced significant reductions in hospital admissions and showed improvements or remission after VNS therapy. Notably, these benefits occurred with right-sided stimulation despite cardiac disease, pacemaker implantation, and pre-existing recurrent laryngeal nerve palsy, without clinically significant bradyarrhythmias or adverse device interactions (6).

A key strength of this report is the systematic description of three complex, real-world patients in whom right-sided VNS was chosen for clearly defined cardiological or anatomical reasons. The series combines detailed longitudinal clinical data, including the course of mood symptoms, ECT exposure, and hospitalizations, with information on stimulation parameters and surgical decision-making. Another strength is the focus on a population that closely reflects the patients most likely to be considered for VNS in routine psychiatric practice: older individuals with chronic mood disorders, multiple prior treatment failures, and extensive medical comorbidities.

In all three cases presented here, the patients responded in mood within one year of VNS activation, leading to complete remission of depressive symptoms. This short delay aligns with the results of recent large-scale studies (7, 8), which reported measurable antidepressant effects within 6 to 12 months after VNS implantation and sustained remission rates afterward, even in patients who had previously failed multiple pharmacological treatments.

Another key point from this case series is that VNS significantly decreased the need for maintenance electroconvulsive therapy (ECT). In two patients, ECT was completely stopped after VNS implantation (Cases 2 and 3) (9). Since about half of all ECT responders relapse within a year, and maintenance ECT can cause cognitive side effects, VNS’s ability to maintain long-term mood stability without repeated anesthesia or hospital stays offers a significant clinical advantage. This aligns with earlier case series and multicenter studies showing that VNS can effectively replace or supplement maintenance ECT in chronic depression (9, 10). Considering the high cost of ongoing ECT treatments, VNS implantation is also likely to be more cost-effective over the long term.

Unlike ECT, the mechanism of VNS likely involves gradual modulation of limbic and autonomic networks. Recent neuroimaging research shows normalization of functional connectivity between the amygdala, hippocampus, and orbitofrontal cortex, along with increased parasympathetic tone and neurogenesis—effects that might support the delayed but lasting antidepressant effects of VNS. The combination of these mechanisms could explain similar therapeutic outcomes for VNS and ECT, despite different neurophysiological pathways.

An additional strength is the multidisciplinary approach to patient selection and follow−up. All three patients were evaluated in joint conferences involving psychiatrists, neurologists, neurosurgeons, and cardiologists, and underwent careful preoperative risk stratification and postoperative monitoring. This reflects current best practice in neuromodulation for complex patients and underscores the feasibility of right−sided VNS when appropriate safeguards are in place.

Beyond its effectiveness in unipolar depression, our data also confirm the positive impact of VNS on bipolar depression, a group that is often underrepresented in controlled trials. In the case presented, VNS provided steady mood stabilization and prevented the recurrence of both depressive and mixed episodes—findings supported by recent studies. These results strengthen the case for VNS as a more widely used neuromodulatory treatment for treatment-resistant affective disorders, not limited to major depressive disorder.

Previously, only a few cases of simultaneous VNS and pacemaker placement have been reported. In Case 3, we demonstrated that the co-existence of VNS and pacemakers in the same patient is also safe and feasible. This conclusion is supported by systematic preoperative testing, intraoperative and titration-phase ECG monitoring, and regular device interrogation during follow-up, none of which revealed any relevant interference. The main concern is that stimulation from VNS devices could be detected by the pacemaker, potentially causing an inappropriate change in pacemaker function. In this case, the patient experienced no issues with pacemaker performance or abnormalities during regular cardiology check-ups.

However, it is essential to perform a precise and thorough assessment of interactions at the maximum VNS output and at the highest sensitivity, particularly in patients with pacemakers.

In the presented case series, there were no significant device-related complications, and temporary issues like mild voice changes or incision-site discomfort resolved on their own. This emphasizes the good tolerability of chronic VNS, consistent with the literature reviewing long-term safety and patient satisfaction. However, right-sided implantation should be carefully monitored, especially for potential cardiac effects. Surgical risks, such as wound-healing complications and vocal cord paralysis, must also be carefully considered when evaluating VNS for individual patients. Additionally, complex medical conditions, as shown in Case 3, require thorough coordination of multidisciplinary care to ensure safety and achieve the best outcomes.

Treatment-resistant mood disorders remain a major therapeutic challenge despite the availability of many pharmacological and somatic treatments. Vagus nerve stimulation (VNS) has become a viable adjunct for patients with chronic, refractory depression and bipolar disorder, as it has been approved for MDD in the US (since 2005) and the EU (since 2020). Recent multicenter prospective studies have confirmed the consistent effectiveness of VNS for depression, showing faster and higher remission rates compared to conventional treatments. Notably, a 5-year follow-up indicated not only sustained symptom improvement, but also lower rates of suicidality and suicide attempts in patients who received VNS.

The complex and longitudinal nature of our case series—with patients experiencing repeated inpatient stays, multiple medication failures, and even cognitive and physical side effects from earlier treatments—underscores the key benefits and challenges of real-world VNS use. All three patients ultimately showed notable improvements in mood stability and had fewer hospitalizations after VNS. Surgical or device-related issues were manageable but emphasize the importance of close multidisciplinary follow-up, especially for patients with severe medical conditions.

Although the effectiveness and tolerability of VNS in our cohort were generally positive, gaps remain in the existing literature regarding very long-term outcomes, optimal patient selection, and the effects of battery depletion or device replacement.

From the patients’ perspective, the possibility of right-sided VNS marked a significant turning point after years of treatment failures, repeated hospitalizations, and severely limited quality of life. Another limitation of this case series is that standardized mood rating scales were not systematically collected at all time points, so our outcome descriptions rely partly on clinical judgment, hospital admission data, and ECT use rather than on fully quantified scale trajectories.

Given the retrospective nature of this case series, the observed associations between right-sided VNS and clinical improvement must be interpreted with caution. Concurrent pharmacotherapy, the natural fluctuation of mood disorders, and potential delayed or cumulative effects of prior ECT may also have contributed to the favorable courses described here.

All three individuals emphasized the importance of achieving a stable mood without ongoing ECT sessions, which had been associated with cognitive side effects, logistical difficulties, and repeated anesthesia. They also expressed relief and reassurance that VNS implantation was possible despite their severe cardiac conditions—factors that had previously limited treatment options and fostered a sense of therapeutic hopelessness.

In follow-up discussions, patients described greater independence in daily life, improved functional ability, and the capacity to resume or maintain work activities. They viewed the right-sided implantation not as a compromise but as a personalized solution that respected their specific anatomical and medical limitations while still providing effective neuromodulation. Collectively, their experiences highlight the importance of individualized, patient-centered decision-making in managing highly refractory affective disorders and the potential of carefully planned right-sided VNS to restore a sense of control, continuity, and long-term hope.

Furthermore, future research and clinical guidelines should consider the economic impact of VNS, including implantation costs and ongoing device maintenance. Larger cohort and registry studies are needed to better understand the long-term effectiveness, safety, and cost-efficiency of VNS for both unipolar and bipolar depressive disorders, as well as to develop strategies for customizing psychiatric neuromodulation.

Our case series supports the growing evidence that VNS is an effective and well-tolerated adjunct treatment for patients with severe, treatment-resistant mood disorders, especially those with complex psychiatric and medical histories. In carefully selected patients, VNS can provide long-term mood stabilization and functional improvement, decrease reliance on inpatient and somatic therapies, and enhance quality of life—offering a valuable alternative for patients with high treatment resistance and chronic dependence on ECT.

## Data Availability

The original contributions presented in the study are included in the article/**Supplementary Material**. Further inquiries can be directed to the corresponding author.
